# P51 Pharmacists as champions in environmental antimicrobial stewardship: reducing antibiotic waste and disposal impact

**DOI:** 10.1093/jacamr/dlag102.057

**Published:** 2026-06-26

**Authors:** Sara Munir, Yasir Riaz

**Affiliations:** Punjab University College of Pharmacy, University of the Punjab, Lahore, Pakistan; University of Agriculture Faisalabad, Faisalabad, Pakistan

## Abstract

**Background:**

Antimicrobial resistance (AMR) is a worldwide health issue of concern, which is not merely triggered by the incorrect use of antibiotics, but also caused by environmental contamination due to improper disposal of unused, unwanted, and expired antibiotics. Pharmaceutical residues that find their way into water bodies, soil and landfills promote the process of selection and spread of antibiotic resistance genes (ARGs) among environmental bacteria, promoting the One Health crisis. Community pharmacists are the most suitable to lead the environmental antimicrobial stewardship campaign (eAMS) by ensuring responsible dispensing, thorough patient education, and the use of safe means to dispose of medications since they have the greatest access to the final chain of medication usage in healthcare delivery.

**Objectives:**

This research evaluated the effects of the eAMS intervention led by a pharmacist on the decrease of the amount of antibiotic waste and the increase of appropriate disposal among patients in community pharmacy.

**Methods:**

A prospective interventional study was done within the chosen community pharmacies in a period of 12 months. Pharmacists were specially trained on eAMS concepts, dispensing of antibiotics in exact quantities, adherence counselling, handling of left-over medicine, and safe disposal measures, such as take-back programmes, deactivation kits. Specific patient education resources were offered to patients on antibiotic therapy, and disposal means were easily available. Structured surveys were used to gather pre- and post-intervention data on the quantity of dispensed and prescribed antibiotics, the volume of antibiotics returned, and the disposal habits of patients using structured surveys and direct measurement of waste. The reduction in active pharmaceutical ingredient (API) load released to the waste stream was measured as the environmental impact.

**Results:**

A total of 1248 patients were enrolled. The eAMS intervention headed by the pharmacist was a significant intervention which resulted in a 52 % (27.6 % to 13.2 % of the dispensing quantity per prescription, *P*<0.001) decrease in the number of antibiotic leftovers, primarily due to customized exact-quantity dispensing and adherence counselling. Pharmacy returns of antibiotics went up by 124 % and the percentage of proper disposal practices went up to 68 %. Unsafe disposal including flushing into toilets or dumping in domestic garbage, dropped immensely by 44% to 12%. Overall, the quantity of the antimicrobial active pharmaceutical ingredient (API) that was safely removed out of the environment was 11.4 kg, it is deactivated, and diverted, which is reducing the risk of pharmaceutical contamination and spread of ARG.

**Conclusions:**

Community pharmacists may be effective proxies of environmental antimicrobial stewardship through incorporation of waste-minimization measures into practice. These are pharmacist-led programmes that will be a viable, sustainable, and inexpensive approach to mitigate the environmental footprint of antibiotics and promote One Health initiatives in AMR. It is highly advised that supportive policies should be used to scale such programmes

